# The "One Portal" Panacea

**Published:** 1901-10-12

**Authors:** 


### THE "ONE PORTAL" PANACEA.

We are not altogether surprised at people now and again poking fun at the medical profession, for indeed there are matters connected with it which savour so strongly of the ridiculous that they are difficult to defend. Among such stands pre-eminent the strange and useless multiplicity of qualifying bodies, and we cannot entirely condemn, or indeed controvert, the savage criticisms of the existing condition of affairs which we hear every year about the time of the opening of the medical schools from bewildered parents vainly endeavouring to master the intricacies of the subject. If there is one thing more than another about which there ought to be neither doubt nor uncertainty it is the standard of knowledge which should be demanded from all candidates for registration as medical practitioners. What then can we think of a system according to which success at any one of upwards of 80 different examinations with innumerable shades of severity is accepted as proof of preliminary education, while the work of examining for actual registration is scattered among twenty-two semi-independent qualifying bodies? All the latter live more or less on the fees extracted from the pockets of the candidates, and may not inaptly be regarded, as we know that by many they are regarded, as fee-sucking parasites of the profession. This is not a case in which it can with any approach to truth be said that there is strength in numbers, for these qualifying bodies are a divided house. The hand of each is against his neighbour. They exist upon fees; fee-producing candidates are to them all as the breath of life, and no very deep knowledge of human nature is required to show that the tendency of the competition between them must be on the "down grade." The whole arrangement is indefensible, and all that can be said in its favour is that like Topsy it "growed."

What then about the remedy which is so often suggested—the "one portal" system, by means of a State examination? At first sight there is much in its favour. The Universities and Royal Colleges could go on giving degrees and diplomas, but the right to practice would depend for all alike upon the State examination, which would fix the minimum standard of knowledge permissible among medical practitioners. We must not imagine, however, that Parliament is ever likely to interfere with the Medical Acts, or pass any laws for the regulation of medical practice, unless it can be shown that such reform is for the advantage of the public quite apart from any benefit which it may offer to the medical profession; and so far as we, as a profession, and medicine as a science, are concerned, the danger of meddling with the Acts as they now stand lies in the conditions which, under the influence of the various quackeries which flourish so greatly in our midst, might be inserted in any new Act under the plea of general public utility. The Medical Acts were passed for the protection of the public and their failure has shown that it is not enough merely to penalise the assumption of medical titles. If the public are to obtain that protection to which they are entitled, something must be done to prevent men acting as medical practitioners until they have passed the proper examinations, and, in the various ways appointed by law, have shown themselves fitted for the work they undertake to do. Any such legislation, however, would evidently be a very serious matter, and would stand on quite a different footing from the existing law. We are very sceptical about Parliament ever permitting any such an interference with free trade in physic, but, and this is the point which we wish to emphasise, if any serious attempt ever should be made to modify the law in the direction of making it a punishable offence for anyone who has not passed a State examination to practice any part of the art of healing (and nothing short of this would really protect the public), then we may be perfectly certain that an equally serious attempt would be made to widen the definition of the word "qualified." We are all of us apt to take one-sided views—doctors not less than most people—and when it becomes a question of deciding who is "qualified" we naturally look at the matter from a professional point of view. But anyone who chooses to mingle with his fellow-men, without proclaiming his profession too obtrusively, must be aware how utterly "graceless" are the public, and people of wealth and position even more than the average, in regard to all questions of medical dogma or professional orthodoxy. Questions which to us appear vital they regard as mere differences of the schools, the voice of the faddist is loud in the land, and if ever the proposal to penalise quackery should be seriously raised, we may be quite sure that the quacks and their following would do their best to widen very considerably the definition of the "qualified practitioner." When, then, we hear the one portal system and the State examination put forward as panaceas for the many anomalies which deface our present regulations, let us not be too ready to snatch at shadows. Logically the present position is absurd. Practically it may be better than reform.

